# The Application of Ultra-High-Performance Liquid Chromatography Coupled with a LTQ-Orbitrap Mass Technique to Reveal the Dynamic Accumulation of Secondary Metabolites in Licorice under ABA Stress

**DOI:** 10.3390/molecules22101742

**Published:** 2017-10-20

**Authors:** Da Li, Guojie Xu, Guangxi Ren, Yufeng Sun, Ying Huang, Chunsheng Liu

**Affiliations:** School of Chinese Materia Medica, Beijing University of Chinese Medicine, Beijing 102488, China; lida.lnzyydx@163.com (D.L.); guojiexu527@gmail.com (G.X.); renguangxiabc@163.com (G.R.); sunyufeng0604@163.com (Y.S.); yinghuang008@outlook.com (Y.H.)

**Keywords:** UHPLC–LTQ-Orbitrap-MS, licorice, secondary metabolites, abscisic acid

## Abstract

The traditional medicine licorice is the most widely consumed herbal product in the world. Although much research work on studying the changes in the active compounds of licorice has been reported, there are still many areas, such as the dynamic accumulation of secondary metabolites in licorice, that need to be further studied. In this study, the secondary metabolites from licorice under two different methods of stress were investigated by ultra-high-performance liquid chromatography coupled with hybrid linear ion trap–Orbitrap mass spectrometry (UHPLC-LTQ-Orbitrap-MS). A complex continuous coordination of flavonoids and triterpenoids in a network was modulated by different methods of stress during growth. The results showed that a total of 51 secondary metabolites were identified in licorice under ABA stress. The partial least squares–discriminate analysis (PLS-DA) revealed the distinction of obvious compounds among stress-specific districts relative to ABA stress. The targeted results showed that there were significant differences in the accumulation patterns of the deeply targeted 41 flavonoids and 10 triterpenoids compounds by PCA and PLS-DA analyses. To survey the effects of flavonoid and triterpenoid metabolism under ABA stress, we inspected the stress-specific metabolic changes. Our study testified that the majority of flavonoids and triterpenoids were elevated in licorice under ABA stress, while the signature metabolite affecting the dynamic accumulation of secondary metabolites was detected. Taken together, our results suggest that ABA-specific metabolite profiling dynamically changed in terms of the biosynthesis of flavonoids and triterpenoids, which may offer new trains of thought on the regular pattern of dynamic accumulation of secondary metabolites in licorice at the metabolite level. Our results also provide a reference for clinical applications and directional planting and licorice breeding.

## 1. Introduction

Licorice is mainly derived from the root of *Glycyrrhiza* species, especially from the root of *Glycyrrhiza uralensis*. Licorice has been widely used in medicine, food, chemicals, animal husbandry, and other fields [1]. China is also one of the world’s largest exporter of licorice with an annual exportation of over 30,000 tons. The annual value of global trade in licorice was estimated as being more than US $42.1 million in 2007 [2,3]. Licorice has been recognized as one of the most famous medicinal plants in traditional Chinese medicine for thousands of years [4]. Flavonoids and triterpenoids are the main secondary metabolites from licorice. They have a wide range of biological activities and have been widely used for the treatment of chronic diseases [5,6,7].

Different external stress factors affect the accumulation of different compounds. Phytohormones, which play a central role in most physiological processes in plants, are becoming increasingly important [8]. Abscisic acid (ABA), a type of sesquiterpenoid phytohormone, is regarded as an important growth inhibitor in plant senescence, cell death, and tissue loss [9]. ABA also plays an important role in enhancing plant resistance to adverse effects and information transmission. Extensive studies have been carried out to examine how ABA plays a role in the regulation of stress responses under drought stress [10]. Furthermore, drought stress can increase the accumulation of secondary metabolites in *G. uralensis*. These results suggest that ABA may play an important role in inducing the accumulation of secondary metabolites [11]. Therefore, using exogenous ABA to stimulate *G. uralensis* (i.e., induce ABA stress) and detecting the change in levels of other components may help to reveal the dynamic accumulation of secondary metabolites in licorice.

Hybrid linear ion trap–Orbitrap (LTQ-Orbitrap) can greatly facilitate the task of metabolite identification. This technique enables parallel data acquisition with high mass accuracy and resolution in addition to providing multistage tandem mass spectrometry (MS^n^) with post-LTQ ion manipulations. In this study, all secondary metabolic analyses were performed on an ultra-high-performance liquid chromatography (UHPLC)-LTQ-Orbitrap, which was coupled with an ESI source. The UHPLC coupled with LTQ-Orbitrap combines the multi-stage mass spectrum function of LTQ and the high-resolution ability of Orbitrap. This can achieve high resolution and multi-level mass spectrometry of the parent ion and daughter ion in a short period of time, which significantly improves the rapid identification of the chemistry of traditional Chinese medicine complex system and analysis of ingredients [12].

A high-performance liquid chromatography (HPLC) method has been developed for studying the chemical components of *G. uralensis* [13]. In this study, UHPLC-LTQ-Orbitrap-MS was utilized to study the secondary metabolites of licorice under ABA stress. The data were analyzed with multivariate statistical credit software. We aimed to understand the dynamic accumulation of the secondary metabolites in licorice, which would be helpful in the directional planting and breeding of *Glycyrrhiza* species.

## 2. Materials and Methods

### 2.1. Plant Material and Reagents

*Glycyrrhiza uralensis* Fisch. were collected from the Ili cultivation base of Xinjiang Province in China and identified by Prof. Chunsheng Liu (Beijing University of Chinese Medicine, Beijing, China). Newly harvested seeds of *Glycyrrhiza uralensis* Fisch. were collected from the identified *Glycyrrhiza uralensis* Fisch. The seeds were submerged in concentrated sulfuric acid for 1.5 h. Following this, the treated seeds were washed with deionized water and soaked in it for 24 h at 25 °C. The seeds were sown in vermiculite in an artificial climate box that was controlled at 25 °C for 16 h light/ 8 h dark cycle. Licorice seeds lasted for 28 days under this treatment. The seeds were divided into 30 samples and cultivated for 28 days. We divided 30 samples into two groups. One group was treated with ABA and the other group was the control group (CG). The plants of ABA-treated group were subjected to treatment with 0.1 mM ABA every 48 h for 28 days [9,14].

All standards were purchased from the CHENGDU MANSITE BIO-TECHNOLOGY CO., LTD (Chengdu, China). Analytical-grade methanol (Tedia, Fairfield, OH, USA) was used for the preparation of sample extractions. Acetonitrile, distilled water and formic acid (Merck, Darmstadt, Germany) of HPLC-grade were used for preparation of the mobile phase.

### 2.2. Sample Processing

We used the roots of *G. uralensis* as testing samples. Samples were carefully washed, frozen (liquid N2), then stored at −80 °C until LC–MS analysis. Samples (100 mg) were accurately weighed, ground, and extracted using an aqueous solution containing 50% methanol and 50% water for 30 min at 4 °C, before the supernatant was collected before testing. Before the analysis, samples were thawed at 4 °C using ultra high purity water, mixed by a vortex and centrifuged at 14,000 rpm for 30 min at 4 °C. Next, the supernatants were analyzed by the UHPLC-LTQ-Orbitrap-MS system [15,16,17]. 

### 2.3. Chromatographic Conditions

Compounds was separated on the UHPLC system and through a phase column (Waters BEH C18 Column with dimensions of 1.7 μm × 2.1 mm × 100 mm, Waters Corp, Milford, MA, USA). The gradient was composed of A (water with 0.1% formic acid) and B (aetonitrile). A constant flow rate of 0.25 mL/min was used with the following optimized gradient: 1% B (0–0.5 min), 1–40% B (0.5–10.0 min), 40–99% B (10.0–11.0 min), 99% B (11.0–12.0 min), and 99–100% B (12.0–15.0 min). The column was maintained at 45 °C at a flow rate of 0.25 mL/min. A 5 μL injection of this sample was inserted into the column [18,19]. 

### 2.4. MS Conditions

All metabolic analyses were performed on a UHPLC-LTQ-Orbitrap MS, which was coupled with an ESI source (Thermo Fisher Scientific, San Jose, CA, USA). The negative polarity mode was applied for compound ionization. The optimized parameters were the following: A capillary voltage of 25 V, an electrospray voltage of 4 kV, a capillary temperature of 350 °C, a sheath gas flow rate of 30 (arbitrary units), an auxiliary gas flow rate of 10 (arbitrary units), and a tube lens of 110 V [18,19,20,21,22,23].

### 2.5. Statistical Analysis

Compared with the standard procedures, the UHPLC-LTQ-Orbitrap-MS data pre-processing includes filter noise, peak recognition, overlapping peak analysis, peak alignment, peak filling, standardization, and normalization. Based on the analysis results, different metabolites were identified. A massLynx software version 4.1 workstation(Waters Corp, Milford, MA, USA) and Xcalibar V2.0 (Thermo Fisher Scientific, San Jose, CA, USA) were used for analysis, while normalized data were examined using SPSS 20.0 and SIMCA-P version 13.0 software (Umetrics, Umea, Sweden) for multivariate statistical analyses. Principal component analysis (PCA), an unsupervised analysis, was performed for the different stresses. PCA, an extremely important approach of multivariate statistical analysis, is widely used for decomposing the 2D matrices, which was set to explain the correlation among numerous variables through a smaller number of underlying factors without losing much information. The peak area of secondary metabolite obtained by UHPLC-LTQ-Orbitrap-MS is standardized by SPSS 20.0. To analyze secondary metabolites, the data were processed to obtain a data matrix containing the samples × variables (samples represent the 30 samples of licorice, variables represent the standardized peak area of secondary metabolites). A supervised partial least squares discriminant analysis (PLS-DA) was employed to compare the differences between different samples of the two groups to identify the key significant compounds. To analyze secondary metabolites, the data were processed to obtain a data matrix containing the samples × variables (samples represent the 30 samples of licorices, variables represent the standardized peak area of secondary metabolites). A typical cross-validation was employed to estimate the number of significant compounds. A permutation test was employed to calculate the validity of the PLS-DA model in terms of overfitting. Compounds with a variable influence on projection (VIP) values greater than 1.0 and a *p*-value below 0.05 were identified as potential biomarkers that could be obtained from the PLS-DA model [24]. Files of data matrices applying PCA and PLS-DA have been added as Supplementary Materials.

## 3. Result and Discussion

### 3.1. Flavonoids and Triterpenes in the Targeted Metabolomics Study

**Figure 1:**
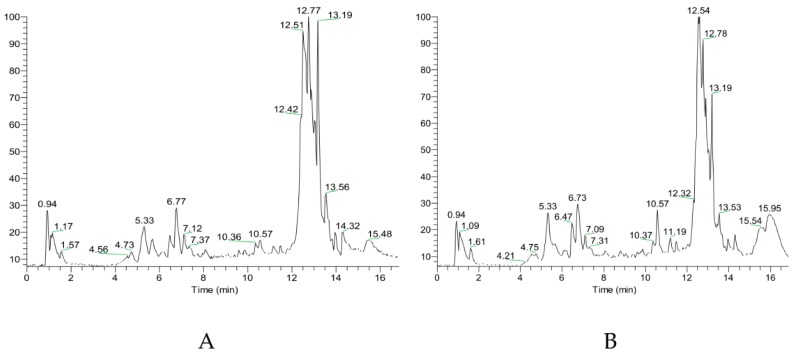
Total ion current chromatogram of UHPLC: (**A**) total ion current chromatogram of samples treated by abscisic acid and (**B**) total ion current chromatogram of control group samples.

In this study, we used the total ion scans and the analysis of flavonoids and triterpenes by multistage mass spectrometry. The peaks in the whole ion scans of licorice samples (ABA-treated and CG groups) were found, while the main components of flavonoids and triterpenes in licorice were identified (Figure 1). Through analysis, a total of 51 compounds were identified, of which 41 were flavonoid pathway metabolites and 10 were triterpenoid saponin pathway metabolites. Seven chalcones were observed: Licoriceglycoside A, licoriceglycoside B, licochalcone A, neolicuroside, isoliquiritin, isoliquiritigenin, and neoisoliquiritin. Seven flavanones were observed: uralenin, naringenin, catechin, liquiritin, liquiritin apioside, liquiritigenin, and glabridin. Five flavonoids were observed: topazolin, trihydroxyflavone, uralene, kaempferol, and luteolin. Eight isoflavones were observed: genistein, gancaonin G, licoisoflavone A, formononetin, licoisoflavone B, semilicoisoflavone B, licoricone, and ononin. Six flavonols were observed: isoquercitrin, neouralenol, quercetin, uralenol, quercitrin, and rutin. Five upstream flavonoids compounds were observed: caffeic acid, ferulic acid, protocatehuic aldehyde, trans-isoferulic acid, and protocatehui acid. Three coumarins were observed: Glycyrol, isoglycyrol, and licopyranocoumarin. Ten terpenoid saponins and their metabolic pathways were observed: Isoglycyrrhizin, glycyrrhizin, licoricesaponin G2, licoricesaponin A3, licoricesaponin B2, licoricesaponin J2, licoricesaponin E2, oleanolic acid, glycyrrhetinic acid, and ursolic acid.

### 3.2. Comparison of Content of Licorice Samples in Two Groups

**Figure 2:**
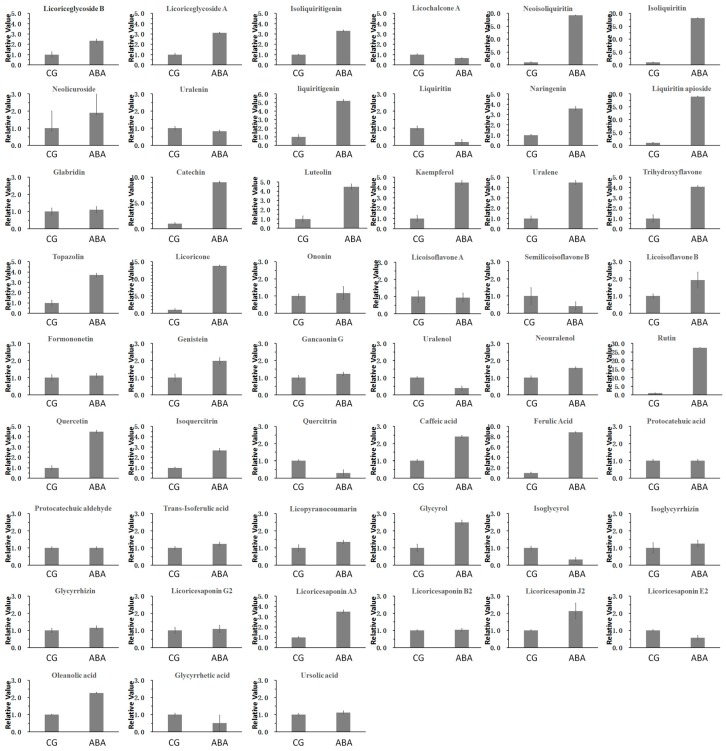
Analysis plot of the contents of 51 components in the two groups of licorice samples. ABA stands for ABA treatment group and CG stands for the control group.

Secondary metabolites are often notably changed under different stresses. This study analyzed 51 major secondary metabolites in licorice (Figure 2). With regard to flavonoids, the results showed that, more than five times, the content was as follows: Neoisoliquiritin, isoliquiritin, liquiritigenin, liquiritin apioside, catechin, kaempferol, licoricone, rutin, and ferulic acid under ABA stress. The results showed that, less than five times, the content was as follows: Licoriceglycoside B, licoriceglycoside A, isoliquiritigenin, neolicuroside, naringenin, luteolin, uralene, trihydroxyflavone, topazolin, ononin, licoisoflavone B, formononetin, genistein, gancaonin G, neouralenol, quercetin, isoquercitrin, caffeic acid, protocatehuic acid, trans-isoferulic acid, licopyranocoumarin, glycyrol licochalcone A, uralenin, liquiritin, licoisoflavone A, semilicoisoflavone B, uralenol, quercitrin, protocatechuic aldehyde, and isoglycyrol under ABA stress.

With regard to triterpenoids, the results showed that isoglycyrrhizin, glycyrrhizin, licoricesaponin G2, licoricesaponin A3, licoricesaponin B2, licoricesaponin J2, and licoricesaponin E2 were increased under ABA stress.

The components of licorice often determine its quality and the amount of its active ingredient is closely related to the ABA stress. To explore the active ingredients of licorice and the relationship between levels of these components and ABA stress, we aimed to determine the influence of licorice flavonoid ingredients and the triterpene composition on the metabolic differences under different stresses. Therefore, we performed a multivariate statistical analysis.

### 3.3. Principal Component Analysis

**Figure 3:**
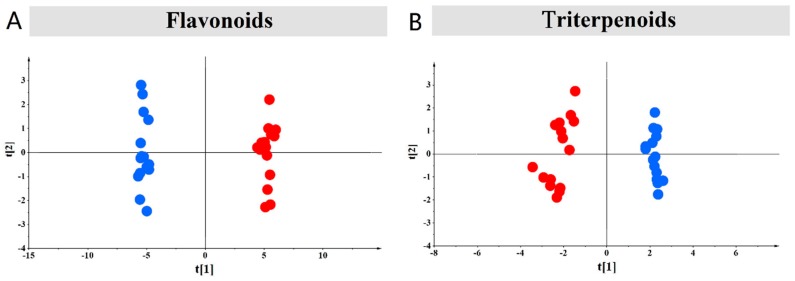
Principal component analysis of ABA-treated and CG samples based on the content of the 41 flavonoids and 10 triterpenoids. (**A**) Represents the PCA of flavonoids; while (**B**) represents the result of triterpenoids. Red points represent the ABA-treated samples, and blue points represent the CG samples.

Principal component analysis, an unsupervised analysis, was performed on the compounds under different stresses. We used an unsupervised pattern recognition analysis method. To analyze flavonoids, the data were processed to obtain a data matrix containing 30 (sample) × 41 (variable). The sample consists of 30 licorice samples, and the variable was the relative peak area of the chemical composition of flavonoids. In the analysis of flavonoids, eigenvalues of more than 1 were extracted, and the three primary components were obtained. All principal components (PCs) had a data variance of approximately 86.6%, while the roles of other PCs were insignificant. From the score map, all the ABA-treated samples were completely distinguished from the CG samples (Figure 3A). To analyze triterpenoids, the data were processed to obtain a data matrix containing 30 (sample) × 10 (variable), before the PCA method was used to analyze these data in order to explain the correlation among numerous variables. The sample included 30 licorice samples, while the variable was the relative peak area of the chemical composition of triterpenoids. For triterpenoids, an eigenvalue of more than 1 was extracted. The main components had a contribution rate of 87.9%. All samples of PCA 3D projections were available. All samples of PCA 3D projections were available. From the score map, all the ABA-treated samples were completely distinguished from the CG samples (Figure 3B).

**Figure 4:**
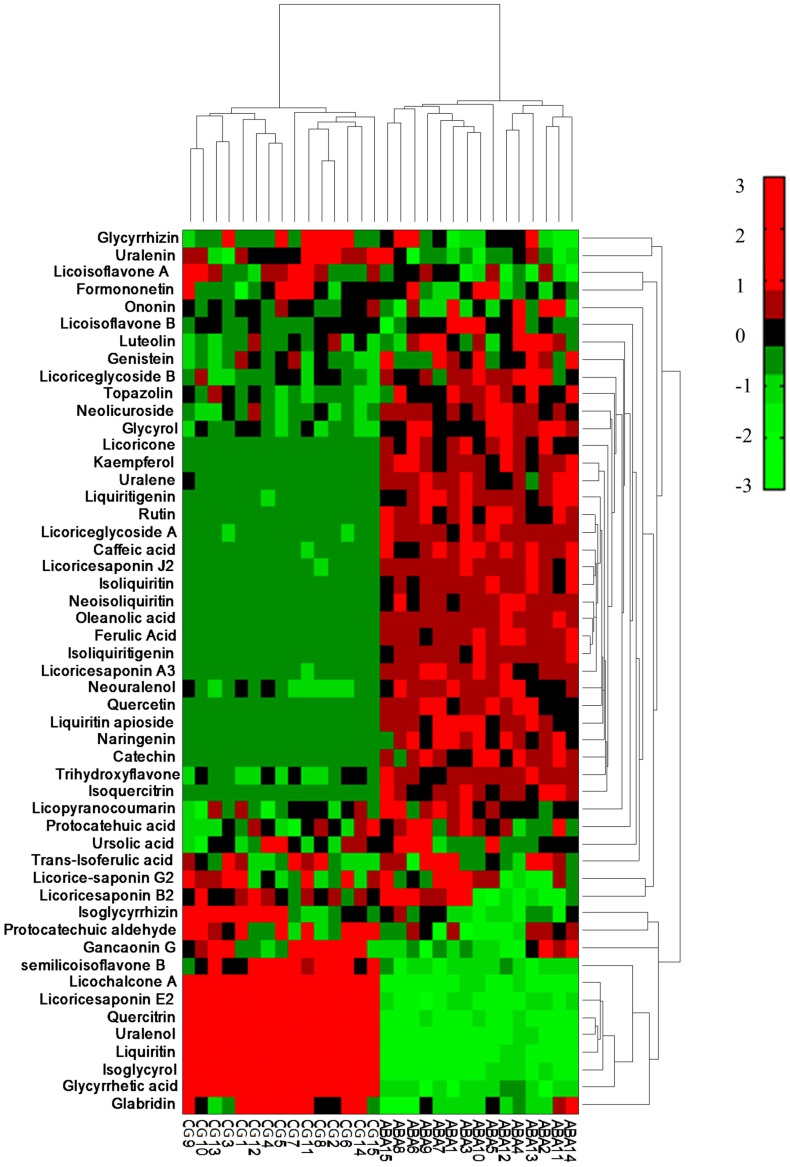
Hierarchical cluster analysis of ABA-treated and CG samples based on the content of the 41 flavonoids and 10 triterpenoids. CG1–CG15 represent the samples of the control group, while ABA1–ABA15 represent the samples of the ABA-treated group.

Hierarchical cluster analysis (HCA) was also assessed in our study. This method provided more reliable and intuitive evidence for understanding differences between ABA-treated and CG groups. The result showed that the samples could be clearly classified into two major clusters with two sub-clusters (Figure 4). The ABA-treated group and CG group were completely separated. However, it was still not clear which major compounds caused the classification of these samples. Therefore, a PLS-DA technique development is necessary to find out definite indexes for describing the differences.

### 3.4. Partial Least Squares Discriminant Analysis

**Figure 5:**
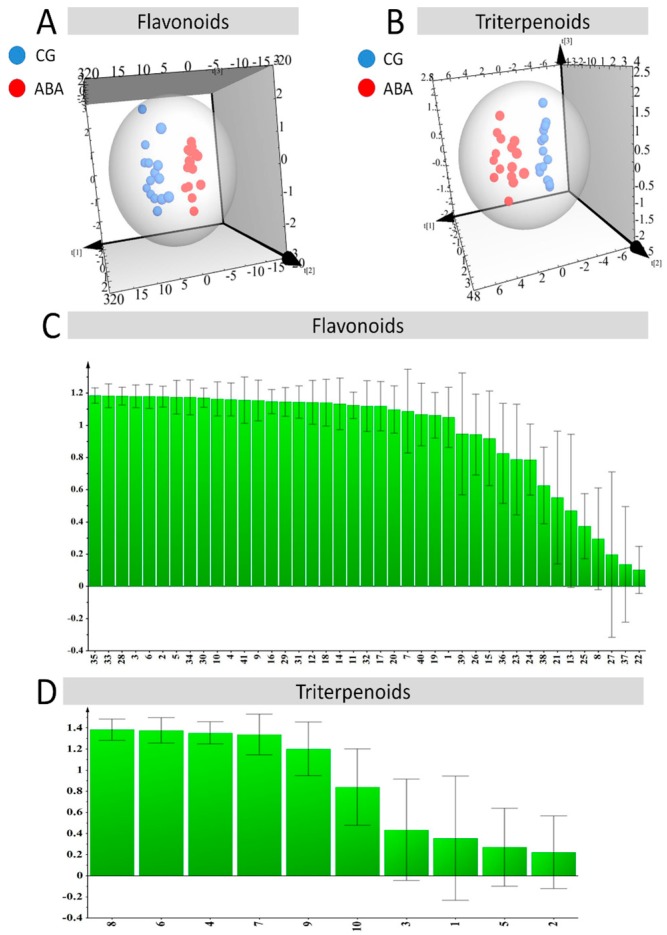
Partial least squares discriminant analysis (PLS-DA) of ABA-treated and CG samples based on the content of the 41 flavonoids and 10 triterpenoids. (**A**) Represents the PLS-DA of flavonoids; while (**B**) represents the result of triterpenoids. Red points represent the ABA-treated samples, and blue points represent the CG samples. Plots C and D show the influence of variables on projection (VIP) diagrams (Numbers in the diagram above: 1. Licoriceglycoside B, 2. Licoriceglycoside A, 3. Isoliquiritigenin, 4. Licochalcone A, 5. Neoisoliquiritin, 6. Isoliquiritin, 7. Neolicuroside, 8. Uralenin, 9. Liquiritigenin, 10. Liquiritin, 11. Naringenin, 12. Liquiritin apioside, 13. Glabridin, 14. Catechin, 15. Luteolin, 16. Kaempferol, 17. Uralene, 18. Trihydroxyflavone, 19. Topazolin, 20. Licoricone, 21. Ononin, 22. Licoisoflavone A, 23. Semilicoisoflavone B, 24. Licoisoflavone B, 25. Formononetin, 26. Genistein, 27. Gancaonin G, 28. Uralenol, 29. Neouralenol, 30. Rutin, 31. Quercetin, 32. Isoquercitrin, 33. Quercitrin, 34. Caffeic acid, 35. Ferulic acid, 36. Protocatehuic acid, 37. Protocatechuic aldehyde, 38. Trans-isoferulic acid, 39. Licopyranocoumarin, 40. Glycyrol, and 41. Isoglycyrol. Numbers in the diagram below: 1. Isoglycyrrhizin, 2. Glycyrrhizin, 3. Licoricesaponin G2, 4. Licoricesaponin A3, 5. Licoricesaponin B2, 6. Licoricesaponin J2, 7. Licoricesaponin E2, 8. Oleanolic acid, 9. Glycyrrhetic acid, and 10. Ursolic acid).

To explore the factors that contribute to distinguishing ABA-treated from CG groups, a PLS-DA analysis of the supervised model was performed [25]. For flavonoids, the data were processed to obtain a data matrix containing the absolute peak area of 30 (sample) × 41 (variable). As shown in Figure 5, ABA-treated and CG samples were significantly discriminated (Figure 5). The values of R2Y and Q2 were 0.99 and 0.95, which indicates that this model may have good fit and predictive ability in data processing. The VIP map, based on the variable projection importance criteria, can reveal the contributing factors that discriminate the samples. The results showed that 9 flavonoids, licoriceglycoside B, licoriceglycoside A, isoliquiritigenin, licochalcone A, neoisoliquiritin, isoliquiritin, neolicuroside, liquiritigenin, and liquiritin, were potential factors that greatly contributed to the identification of the ABA-treated samples.

For triterpenoids, the data were processed to obtain a data matrix containing the absolute peak area of 30 (sample) × 10 (variable). As shown in Figure 5, ABA-treated and CG samples were significantly different (Figure 5). The values of R2Y, and Q2 were 0.99 and 0.97, which indicates that this model may have a good fit and predictive ability in data processing. The results showed that the 9 flavonoids, including licoricesaponin A3, licoricesaponin J2, licoricesaponin E2, and oleanolic acid, were potential factors that greatly contributed to the identification of ABA-treated samples.

Briefly, our results presented that ABA stress could significantly change the content of secondary metabolites in licorice and generally contribute to the accumulations of secondary metabolites, which suggest that ABA can be an excellent regulator for directionally planting licorice [26,27].

### 3.5. Analysis of Metabolic Pathways of Two Compounds

**Figure 6:**
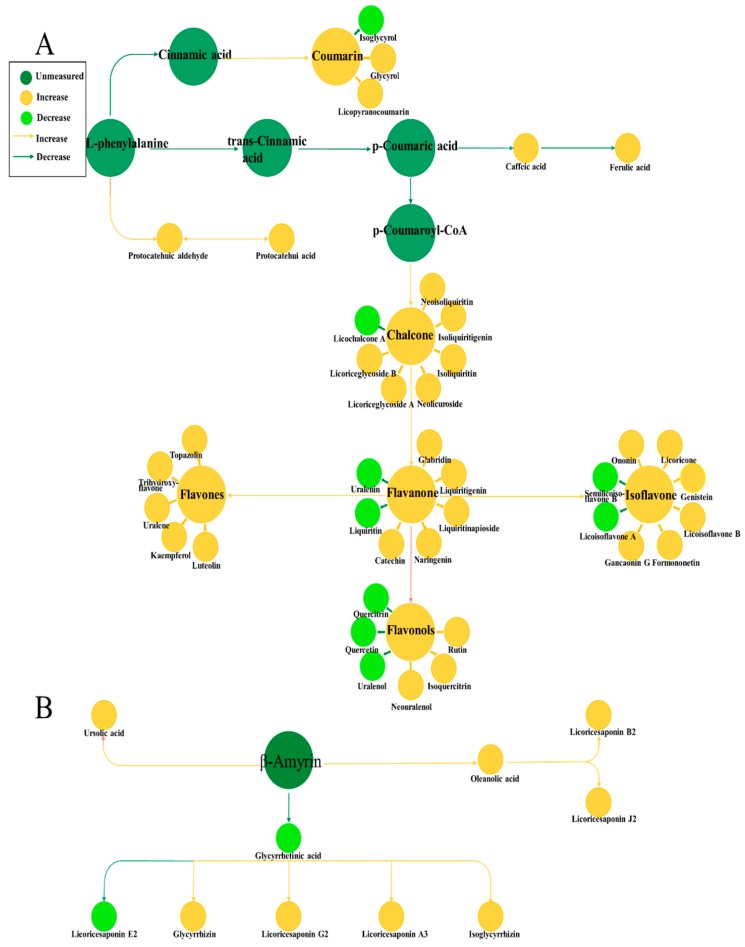
Visualization of secondary metabolite dynamics in a biochemical pathway map. (**A**) represents the pathway map of flavonoids; while (**B**) represents the result of triterpenoids. Dark green circles represent unmeasured components, green circles represent decreased components, and yellow circles represent increased components.

To comprehensively understand the dynamic accumulating pattern of metabolites, all the characterized secondary metabolites in licorice were integrated and analyzed with corresponding pathways. As shown in Figure 6, the overall metabolites that increased in the biosynthetic pathway of flavonoids were coumarins, chalcone, flavanones, flavone, flavonols, and isoflavones. Specifically, the compounds that had increases in content included the following: coumarins, such as glycyrol and licopyranocoumarin; chalcones, such as licoriceglycoside B, licochalcone A, neolicuroside, isoliquiritin, isoliquiritigenin, and neoisoliquiritin; flavanones, such as naringenin, catechin, liquiritin apioside, liquiritigenin, and glabridin; flavones, such as topazolin, trihydroxyflavone, uralene, kaempferol, and luteolin; isoflavones, such as genistein, gancaonin G, formononetin, licoisoflavone B, licoricone, and ononin; and flavonols, such as isoquercitrin, neouralenol, and rutin. Specifically, compounds that had decreases in content included the coumarins, such as isoglycyrol; chalcones, such as licochalcone A; flavanones, such as uraleni and liquiriti; flavonols, such as quercitrin, quercetin, and uralenol; and isoflavones, such as semilicoisoflavone B and licoisoflavone A.

In the biosynthetic pathway of triterpenoids, glycyrrhetinic acid and licoricesaponin E2 decreased, while licoricesaponin A3, glycyrrhizin, isoglycyrrhizin, licoricesaponin G2, licoricesaponin B2, licoricesaponin J2, oleanolic acid, and ursolic acid increased. In general, our results showed that most secondary metabolites in licorice increased under ABA stress. This provided a comprehensive dynamic pattern of secondary metabolites, which is helpful for us to understand the way they accumulate.

Glycyrrhizin is one of the most important secondary metabolites in licorice. They are generally accumulated during certain periods of development and are sensitive to changes under external stresses. Recently, our research group found one unique *Gu*UGAT, which was able to catalyze the continuous two-step glucuronidation of glycyrrhetinic acid to directly yield glycyrrhizin. Thus, the complete pathway of glycyrrhizin biosynthesis was determined [14]. In our study, the content of glycyrrhetinic acid in licorice decreased, but the content of glycyrrhizin increased. This change may result from differences in the expression of key genes and enzymes, which lead to changes in the accumulation rates of secondary metabolites. Therefore, integrated analysis of the key genes involved in the pathways may help to further reveal the mechanisms of dynamic changes in the secondary metabolites from licorice [28,29,30,31,32,33].

ABA is an important signal transduction molecule in plants and plays an important role in the regulation of plant resistance. It had been reported that the content of glycyrrhizin in licorice has an obvious correlation with the content of ABA under drought stress. Those studies indicate that there are some connections between glycyrrhizin and ABA. Consistently, this study revealed the dynamic accumulation of glycyrrhizin in licorice under ABA stress, which further confirms this type of connection [34,35].

It may raise a question: how does ABA connect with glycyrrhizin? We think that it may be related to the inhibiting effects of internal ABA in its biosynthetic pathway. It had been reported that the application of exogenous ABA can supplement the endogenous ABA, so that the concentration of ABA in the leaves is relatively increased [9]. ABA and glycyrrhizin result from the same 2-*C*-methyl-Derythritol-4-phosphate (MEP) and mevalonate (MVA) pathways, but their pathways are separated in the node of isopentenyl pyrophosphate (IPP). Therefore, the reason that ABA is able to induce glycyrrhizin may be that the exogenous ABA interferes with the biosynthesis of internal ABA, thus allowing more IPPs to flow into biosynthetic pathway of glycyrrhizin.

## 4. Conclusions

There is hormone-specific comprehensive reprogramming of secondary metabolites in the economically important licorice under different types of stress. For the first time, the dynamic accumulation of secondary metabolites in licorice under ABA stress was analyzed by UHPLC-LTQ-Orbitrap-MS. The complicated metabolic pattern was related to two hormone-specific planting districts from ABA-treated group and control group for licorice. The targeted results showed that the components of flavonoids and triterpenoids were different in ABA-treated group, with different accumulation patterns of different compounds. The main potential compounds that accumulate in licorice under ABA stress were determined in our research. The data can provide a reference for the directional breeding of licorice. This study includes a better approach to exhibiting the association between the accumulation of secondary metabolites and specific hormones in licorice, which is useful for identifying potential biologically relevant compounds.

## Supplementary Materials

Supplement materials are available online.
